# Prevalence of Depression among Households in Three Capital Cities of Pakistan: Need to Revise the Mental Health Policy

**DOI:** 10.1371/journal.pone.0000209

**Published:** 2007-02-14

**Authors:** Amin A. Muhammad Gadit, Gerry Mugford

**Affiliations:** 1 Discipline of Psychiatry, Memorial University of Newfoundland, St. John's, Newfoundland, Canada; 2 Faculty of Psychiatry, Pharmacy and Medicine, Memorial University of Newfoundland, St. John's, Newfoundland, Canada; Cornell University, United States of America

## Abstract

**Background:**

Pakistan, among the other developing countries, has a higher prevalence rate of depression because of the current social adversities. There is, thus, a great need for systematic studies on prevalence of depression. The current study aims at exploring the prevalence of depression among households in three capital cities of Pakistan.

**Methodology and Principal Findings:**

A sample of N = 820 was randomly selected, and a cross sectional telephone-based study was conducted for a duration of six months. It was found that there was a regional variation in prevalence rates for depression among the three cities. Lahore had the highest number of depressives (53.4%), as compared to Quetta (43.9%) and Karachi (35.7%). Middle age, female gender and secondary school level of education were significantly associated with depression among the study group.

**Conclusions/Significance:**

The different rates of prevalence among the three cities could be attributed to local cultural influence, geographical locations and social adversities. There is a need for revision of existing health policy by the government.

## Introduction

Depression has been recognized as a major public health problem evidenced by its ranking of fourth position among the global burden of diseases. Many believe it will it will occupy second position by the year 2020. 340 million people above the age of 18 suffer from depressive disorders that contribute to a high suicide rate. [1]

450 million people in the world suffer from a mental or behavioral disorder. W.H.O. (World Health Organization) global burden of diseases, 2001, states that 33% of the years lived with disability (YLD) are due to neuropsychiatric disorders, unipolar depressive disorders alone lead to 12–13% of years lived with disability and rank as the third leading contributor to the global burden of diseases[2].

Depressive disorders were estimated to be the leading cause of disability in the world in 1990, accounting for 10.7% of total YLD. These disorders are the 4^th^ leading cause of total DALYs (3.7% total disability adjusted life years) [3]. Estimated global deaths due to unipolar depressive disorders was 12,044, with 5462 male and 6582 female (2004) [4]. A large study conducted by WHO in fourteen countries showed 24% primary care attenders worldwide received an ICD-10 psychiatric diagnosis, the most common of which was ‘current depressive episode’ [5].

In developing countries 10–44% suffers from depression and anxiety disorders, less than 35% receive care and according to an estimated 50.8 million people suffer from major depression [6].

**Figure 1:**
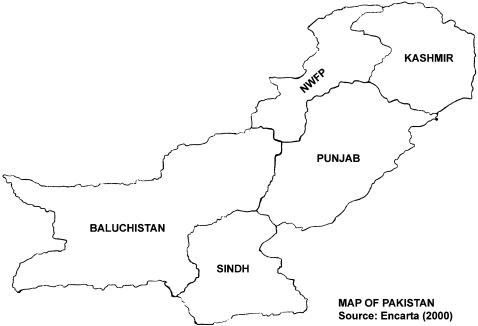
Map of Pakistan.

Pakistan is the 9^th^ most populous country in the world, though area wise it ranks thirty-fourth among the thirty-seven low-income countries (Figure 1). It is the fourth most populous country after Bangladesh, China and India. It has total population of 157,935000, GDP (gross national product) per capita (intl $ 2004) 2,151, life expectancy at birth m/f: 62/63, infant mortality: 102/1000, total health expenditure per capita (intl $ 2003): 48, total expenditure as % of GDP: 2.4, GNI (gross national income) per capita is $600, urban population: 34%, literacy rate: 49% [7], population below poverty line [8]: 35%, total number of physicians is 128073 which includes 18, 633 specialists [9]. The total number of psychiatrists is 250 and for such a large population this is a seriously insufficient number. (Table 1)

**Table 1:** Vital information about Pakistan

Population of Pakistan	157,93,5000
GDP (gross national product)/capita	2,151
Life expectancy at birth	m/f: 62/63
GNI/Capita	$600
Urban population	34%
Literacy rate	49%
Population below poverty line	35%
Total number of physicians	128073
Total number of psychiatrists	250

The magnitude of mental illness is: 6% depression, 1.5% schizophrenia, 1% Alzheimer's disease, 1–2% epilepsy and the other disorders [10]. This current situation in Pakistan along with other basic health problems, the social upheaval, political instabilities, lawlessness, terrorism, economical disparity, problems with security and safety has created a ground fertile for depression which has almost taken first position among the all psychiatric conditions. A number of different studies mainly clinic based report varying rates for prevalence of depression. According to a country profile report by WHO Emro region, 10–16% of general population in Pakistan suffers from mild to moderate psychiatric illnesses in addition to the 1% suffering from severe mental illnesses [11]. Extrapolation of prevalence rates for depression in Pakistan yields approximately 8,437,406 out of the 157,935,000-population figure [12]. Mirza and Jenkins [13] made a systematic review of published literature that gave prevalence estimates for depression and anxiety and discussed associated risk factors. In their review, they found the mean overall prevalence of depressive disorders and anxiety was 34% (range 29–66% for women and 10–33% for men). Factors associated with anxiety and depressive disorders were female sex, middle age, low level of education, financial difficulty, being a housewife and relationship problems. In a community based psychiatric clinic among all the patients who were seeking psychiatric help, 47% were found to be suffering from depression [14]. This study was conducted retrospectively on N = 700 patients who attended the psychiatric clinic during 1995–1997. The criterion for diagnosing depression was based on ICD-10. Based on other comprehensive local clinic based studies in all provinces of Pakistan, the findings regarding prevalence of depression were: Sindh: 16% urban, 12% rural, Punjab: 8% urban, 9% rural, Baluchistan: 40% urban, 2.5% rural, NWFP: 5% urban, 3% rural [15]. In a community-based study [16] conducted on an island of Karachi among adult women belonging to fisherman community, the point prevalence of depression was 7.5%. The criterion for diagnosing depression in this study was based on using Mini International Neuropsychiatric Interview, which was supplemented by ICD-10. In another study conducted in Punjab, the adjusted prevalence of depressive disorders was 44.4%: 25.5% in males and 57.5% in females [17]. This study was a two-phase survey in a village which was done with the help of Personal Health Questionnaire (PHQ) and the self rating questionnaire (SRQ). Many of the studies were conducted in urban centers probably because of the available psychiatric set-ups and hence prevalence rates are geared towards urban dominance. Though few studies were conducted on population, many were clinic based, and hence there is an identified need for more population based studies. Additionally, none could be found which show province wide differences keeping in view the cultural, linguistic and traditional background of people living in these provinces. The current study aims at looking at the morbidity patterns of three main provincial capital cities of Pakistan in terms of prevalence of depression.

## Methods

### Participants and Procedures

#### Study location

A telephone based survey study was conducted in Pakistan with a view to cover all four provinces. The capital cities of three provinces: Sindh, Punjab and Baluchistan, which are Karachi, Lahore and Quetta, were selected for the study. Peshawar, the capital of NWFP was not included due to the turbulent situation resulting from the earthquake devastation.

#### Sample size

For calculation the frequency of depressed diagnosed individuals in Quetta was taken as 9% with population of 300,000, the level of significance (α) equal t o 0.05 and bound of error 5% (deviation from the actual value), the estimated sample size calculated for the given prevalence at 95% confidence interval: 200.

For calculation the frequency of depressed diagnosed individuals in Karachi was taken as 8.6% with population of 10 million, the level of significance (α) equal t o 0.05 and bound of error 5% (deviation from the actual value), the estimated sample size calculated for the given prevalence at 95% confidence interval: 236.

For calculation the frequency of depressed diagnosed individuals in Lahore was taken as 50%, population of 6,563,000, the level of significance (α) equal t o 0.05 and bound of error 5% (deviation from the actual value), the estimated sample size calculated for the given prevalence at 95% confidence interval: 384. A sample size of N = 820 was thus determined.

The discrepancy in the frequency is due to the different prevalence rates in three different cities gathered from available government record. Three different cities were explored separately and through the computer program, the sample sizes were estimated.

#### Procedures

A total of 820 households were selected using a telephone book. A random number was assigned i.e. every 3^rd^ number was picked and a telephone call was made to ascertain the presence of a household member with depression. This was determined by a screening question: “Have you or has any one in your house hold ever been diagnosed with depression or depressed mood by a doctor?” If the answer was negative, only the demographic details were noted. If the answer was “yes”, then a questionnaire-based survey was conducted inquiring about illness. Interviews were conducted in Quetta (N = 200), Karachi (N = 236) and Lahore (N = 384). The number of interviews on phone was according to the statistically derived sample size, the process of interviews continued until that number in each city was achieved keeping in account the refusals met for being interviewed on phone. The person who answered the phone was inquired about whether he/she was suffering from depression or any other member of the household had this problem. If there was information about someone else in the household who was the sufferer then the request was made for a direct talk with the concerned person. No proxy information was noted. The household size was not taken into consideration in the multivariable analysis. Generally, those who refused the interview were not included. The differences in the types of household members among those who refused and those who agreed to be interviewed were not recorded. The independent variables in the study were city, age, sex and education and the dependent variable was depression. The independent variable education was broken down to different components, like: illiterate (no formal education at all), primary (grades 1–5), secondary (grades 6–10), matriculated (passed grade 10^th^), graduate (the bachelor education which is grade 14), postgraduate (above bachelor's education i.e. masters, doctorate). The dependent variable ‘depression’ was diagnosed/assessed on the basis of predominant depressed mood for at least two weeks on a continuous basis and any five of the following symptoms: insomnia or hypersomnia, loss or increase of appetite, lack of concentration, problems with memory, lack of energy, fatigue, weight loss/gain, excessive worry, guilt, muscle aches, irritability, loss of pleasure, hopelessness, death wishes or suicidal ideation, weeping tendencies, loss of interest and lack of libido. It was also asked whether any of these symptoms affected life routine, family life or study/employment.Primary responders were those who answered the phone and were depressed themselves whereas secondary responders were those who initially did not answer the phone but came to the phone for interview upon request. If there was no one in the house hold with depression, then only demographic information was obtained from the person who answered the phone. The details about the study were conveyed to each interviewee and confidentiality was assured. The total duration of the study was six months, starting date: January, 2006 to June, 2006.

##### Analysis

SPSS version: 14.0 were used for the analysis.

##### Ethics Committee Approval

Before the conduction of this study, questionnaire and proposal was presented to the ethics committee of Hamdard University, Karachi-Pakistan who after careful review approved the study.

## Results

**Figure 2:**
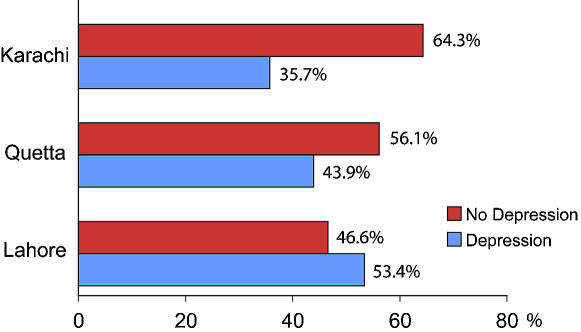
Showing frequency of depression as compared to no depression in the three cities of Pakistan.

The results show that out of 820 people interviewed or contacted, 45.98% reported suffering from depression or having symptoms of depression. Among the three cities, Lahore had the maximum number of respondents: (Table 2) 46.8%, as compared to Quetta (24.1%) and Karachi (29.0%). 57% of the respondents were males and 43% were females. 2.7% were illiterate and 18.9% were postgraduates, other categories were lying in between primary to graduate level of education. The majority of sufferers (57%) had secondary level education. 36.7% were the primary responders who had depression whereas, 63.3% were secondary responders. As compared to Karachi (35.7%), Lahore had the highest number of depressives (53.4%) with p = 0.0003 and adjusted odds ratio 1.89 (1.33, 2.68) followed by Quetta (43.9%) with p = 0.0033 and adjusted odds ratio of 1.91 (1.24, 2.94), (Figure 2). The mean age of depressives was 36.8 yr (CI 34.5, 38.1), with females reporting higher rates of depression (51%), (Table 3). On univariable analysis, the association of self reported depression included older age, female sex, residence in Quetta or Lahore and level of education. On multivariable analysis, the associations of self-reported depression included older age (adjusted odds ratio AOR 1.02 per year), female sex (AOR 1.53), and residence in Quetta (AOR 1.91 vs. Karachi) or Lahore (AOR 1.89 vs. Karachi), the level of education was of border line significance. The results in table 2 are not exclusive to primary responders or those with depression only.

**Table 2:** Population characteristics of (N = 820)

Depression %	
No	54.02
Yes	45.98
City %	
Karachi	29.0
Quetta	24.1
Lahore	46.8
Age (years), Me (SD)	35.0 (13.1)
Sex %	
Male	57.0
Female	43.0
Education %	
Illiterate	2.7
Primary	9.5
Secondary	13.0
Matriculated	28.8
Graduate	26.7
Post-graduate	18.9
Other/missing	0.4
Primary responder %	
No	63.3
Yes	36.7

**Table 3:** Comparison of subjects with and without depression/depressive symptoms

	No Depression	Depression	P	AOR	P
City (%)			<.0001		
Karachi	64.3	35.7	χ^2^ = 48.91, df. = 2	1 (ref )	–
Quetta	56.1	43.9		1.91 (1.24,2.94)	0.0033
Lahore	46.6	53.4		1.89 (1.33,2.68)	0.0003
Age (years)	33.5 (32.4,34.6)	36.8 (34.5,38.1)	0.0002 F = 13.88, df. = 1	1.02 (1.01–1.04)	0.0007
Sex (%)			0.012		
Male	57.8	42.2	χ^2^ = 6.28, df. = 1	1 (ref)	–
Female	49.0	51.0		1.53 (1.13,2.07)	0.0058
Education			<0.0001		
Illiterate	0	100.0	χ^2^ = 41.75, df. = 6	-inestimable	–
Primary	53.8	46.2		1 (ref)	–
Secondary	43.0	57,0		1.98 (1.08,3.66)	0.0284
Matriculated	55.9	44.1		1.08 (0.63,1.83)	0.7904
Graduate	54.3	45.7		1.09 (0.64,1.88)	0.7485
Post-graduate	65.2	34.8		0.72 (0.41,1.28)	0.2635
Other/missing	100.0	0.0		inestimable	–

* Adjusted for City, Age, Gender and Education

## Discussion

In this study, the overall rate of depression was 45.98%; this includes all people reporting a confirmed diagnosis of depression or with features of depression. This figure is similar to another study conducted in a community-based clinic of Karachi, which showed 47% of patients attending psychiatric clinic suffered from depression [14]. A study [13] by Mirza and Jenkins reports prevalence rates of 34% for depression as well as anxiety. Mumford [18] found 66% of women and 25% of men in two rural areas to be suffering from depression. Ahmad et al [19] found in another rural sample found that 72% of women and 44% of men were suffering from anxiety and depression. Husain et al [17] found adjusted prevalence for depressive disorder to be 44.4%. This rate in the current study is consistent with the general figure for developing countries where 10–44% of people suffer from depression and anxiety [6]. This contrasts sharply with the 6% prevalence rate figure for depression over all which may be an extrapolation from figures available from various sources from national database [10]. In view of a poorly developed information management system in Pakistan and flawed method for collecting statistics, strong emphasis cannot be placed on this overall figure. However, there is an identified need for collecting nationwide data in order to understand the magnitude of this problem in true sense. The mean age of sufferers was 36.8 years in the current study, which is close to another local study in which the mean age was 34.31 years. In the study by Mirza and Jenkins [13], association with depression was found with middle age and onwards. There is a linear rise with age in the mean score and prevalence of self reported symptoms of depression [20]. Age- related decline in central serotonergic function might make older individuals more vulnerable to depression and possibly render depressive episodes more frequent with increasing severity and being less amenable to treatment [21]. In a study [22] it was described that depression reaches its lowest level in the middle age at about age 45, rise in late life reflecting life cycle gains and losses in marriage, employment and economic well being. The issue of what is called middle age is interesting. Local definitions quote a range from 40–59 years as ‘middle age’, however, the argument can be put forward as to what is called ‘middle age’ may vary according to the measured life expectancy at birth in different countries. In Pakistan, the life expectancy at birth for males and females is 61 and 63 years respectively, in this regard, the mean age of sufferers may qualify for being in this category.

Females outnumber males as sufferers in the current study, which is consistent with other studies [14], [17]. In the present study, secondary level of education was associated with depression and this was observed by others [14]. The striking feature in this study is the differential rates of depressive disorder prevalence in the three cities. It is important to bear in mind that all these three cities have different cultural background, have different local languages and different traditions. An interviewer bias cannot be ruled out when it comes to linguistics, expressions and concepts. People in Lahore are generally more expressive, have wider exposure to psychiatric services but rates of unemployment and poverty are also relatively high with high number of population and overcrowded conditions. Quetta is a city of low expression, strong traditional bindings, cultural inhibitions and limited availability of resources. High prevalence of depression can possibly be explained on this basis. Surprisingly, Karachi, which has become hub of violence, terrorism, lawlessness, economic disparity, huge population and great safety issues, has shown relatively low prevalence of depression. This can be explained on the basis of learned helplessness, which has overcome the inhabitants who are exposed to adversities for more than two decades. The higher general prevalence of depression has also been supported by a previous study [17] on the grounds of social adversity.

The mental health policy was first formulated in 1997, which addressed issues of advocacy, promotion, prevention, treatment, rehabilitation and intersectoral collaboration. It envisaged to train primary care providers, to establish resource centers at teaching hospitals and psychiatric and detoxification centers. There was provision for crisis intervention and counseling services, special facilities for mentally handicapped and up gradation of large mental hospitals. The allocated mental health budget is 0.4% of total health care expenditures. As such the policy for mental health is not comprehensive and do have multiple lacunae. It does not address the issues related to morbidity patterns. As such, there is a dire need to revise the mental health policy in order to address the mental health issues effectively and appropriately.

The present study has some limitations: narration of cluster of symptoms rather than discreet entities, cultural bias, interviewer style, interviewer-bias and the inherent problem of cross-sectional studies.

### Conclusion

The prevalence of depression was found to be different in three capital cities of Pakistan, which could be due to local cultural influence, geographical locations and social adversities. It is suggested that the government should reconsider revising the existing mental health policy and adopt a differential approach in addressing the varying regional magnitude of mental health morbidity.

### Suggestions

- Data collection system of the country needs improvement.
- Development of efficient Management Information System in the country.
- Need for a nation-wide study to assess the real magnitude of mental health morbidity.
- Trend for compilation of vital statistics should be promoted.

## Supporting Information

Questionnaire S1 Depression Questionnaire - Pakistan
(0.03 MB DOC)
