# Supplementary material for: Prevalence of Depression among Households in Three Capital Cities of Pakistan: Need to Revise the Mental Health Policy
Source: PLoS One. 2007 Feb 14;2(2):e209. doi: 10.1371/journal.pone.0000209 (PMC1790700; doi:10.1371/journal.pone.0000209)
Supplement: Questionnaire S1 — Depression Questionnaire - Pakistan (0.03 MB DOC) [file pone.0000209.s001.doc]

QUESTIONNAIRE ON:

**“PREVALENCE OF DEPRESSION AMONG HOUSEHOLDS IN THREE CAPITAL CITIES OF PAKISTAN: NEED TO REVISE THE MENTAL HEALTH POLICY”**

Date:

Serial Number: Location:

Computer code number:

1. **Have you or has anyone in your household ever been diagnosed with depression or a depressed mood by a doctor?**

None----------

1 Person---------

2 Persons---------

More than 2 -------------

How many non-sufferers? --------------

2- A-Sufferer: Age-------- Sex----------- Education

Illiterate------

B-Non-sufferer: Age: ---- Sex: ---- Primary------

Secondary--------

Matriculated-----

Graduate------

Postgraduate----

Other---------

If suffering from depression, then the duration: ----------------------------------

3-**Do you suffer from any of these?**

Depressed Mood --------------

Fatigue/Lack of energy ---------------

Loss of Interest ----------------

Loss/increase in appetite --------

Irritability --------------------

Problems with memory -------

Impaired Concentration ---------------

Excessive Worry -----------------------------

Guilt --------------------------------

Sleeping too much ------------------------------------

Insomnia -----------------------------

Suicidal Ideation -----------------

Muscle Aches ------------------------

Loss of libido------------------

Loss of weight/increase in weight---------------

Hopelessness ----------------

Loss of pleasure -------------

Weeping episodes -----------

Other symptoms-------------

4**- Does the above symptoms interfere with your life routines, family life and study/employment?** Yes------ No -------
